# Human-in-the-Loop Trajectory Optimization Based on sEMG Biofeedback for Lower-Limb Exoskeleton

**DOI:** 10.3390/s24175684

**Published:** 2024-08-31

**Authors:** Ling-Long Li, Yue-Peng Zhang, Guang-Zhong Cao, Wen-Zhou Li

**Affiliations:** 1 Guangdong Key Laboratory of Electromagnetic Control and Intelligent Robots, College of Mechatronics and Control Engineering, Shenzhen University, Shenzhen 518060, China; 2150296002@email.szu.edu.cn (L.-L.L.); liwenzhou@email.szu.edu.cn (W.-Z.L.); 2 Shenzhen Institute of Information Technology, Shenzhen 518172, China; ypzhang@sziit.edu.cn

**Keywords:** lower-limb exoskeleton, human-in-the-loop, motion planning, human–machine system

## Abstract

Lower-limb exoskeletons (LLEs) can provide rehabilitation training and walking assistance for individuals with lower-limb dysfunction or those in need of functionality enhancement. Adapting and personalizing the LLEs is crucial for them to form an intelligent human–machine system (HMS). However, numerous LLEs lack thorough consideration of individual differences in motion planning, leading to subpar human performance. Prioritizing human physiological response is a critical objective of trajectory optimization for the HMS. This paper proposes a human-in-the-loop (HITL) motion planning method that utilizes surface electromyography signals as biofeedback for the HITL optimization. The proposed method combines offline trajectory optimization with HITL trajectory selection. Based on the derived hybrid dynamical model of the HMS, the offline trajectory is optimized using a direct collocation method, while HITL trajectory selection is based on Thompson sampling. The direct collocation method optimizes various gait trajectories and constructs a gait library according to the energy optimality law, taking into consideration dynamics and walking constraints. Subsequently, an optimal gait trajectory is selected for the wearer using Thompson sampling. The selected gait trajectory is then implemented on the LLE under a hybrid zero dynamics control strategy. Through the HITL optimization and control experiments, the effectiveness and superiority of the proposed method are verified.

## 1. Introduction

Lower-limb exoskeletons (LLEs) are advanced wearable robotic systems that assist lower-limb movements through integrated mechatronic systems and sensing and control networks [1,2,3,4]. The LLEs can offer rehabilitation training and walking assistance for individuals with lower-limb dysfunction or those in need of functionality enhancement in a human–machine interaction manner, presenting a wide range of application prospect [5,6,7]. Motion planning plays a significant role in LLE control, as it directly impacts human performance while wearing the exoskeleton. Consequently, motion planning has emerged as a prominent yet challenging issue in robotics [8,9]. Among the various strategies employed for motion planning, trajectory optimization is particularly noteworthy. It aims to generate an optimal motion profile that can be precisely executed by the controller, thereby ensuring smooth and efficient movement [10,11,12]. However, most LLEs have not fully considered the dynamic and the complex motion characteristics of human users in trajectory optimization [10], leading to subpar human performance [13].

Human-in-the-loop (HITL) optimization has emerged as a promising approach to address this challenge. The concept of HITL was initially introduced by Collins et al. in 2017 [13]. The HITL optimization involves incorporating human feedback into a certain decision-making process within the iteration of the optimization algorithm. This approach functions as a “black box” within a human–machine system (HMS), enabling automatic and heuristic personalization of assistance machine tailored to the specific needs of the user [14,15]. In [13], the HITL optimization was utilized in an ankle exoskeleton to identify an assistance control strategy aimed at minimizing metabolic cost during walking, the objective was to search for the optimal torque pattern within a single-gait cycle. The ankle torque was defined with respect to four independent parameters, and the cost function of metabolic cost was derived by respiratory data. Then, through the application of derivative-free optimization using a covariance matrix adaptation evolution strategy (CMA-ES), the optimal parameter values were iteratively determined. Building on this methodology, Song et al. [16] employed a similar HITL approach to identify the optimal torque curve that facilitated the ankle exoskeleton to assist with faster self-selected walking. Gordon et al. [5] further advanced the field by utilizing musculoskeletal modeling to assess the simulated metabolic rate in real time, thereby optimizing the assistive torques provided by a hip exoskeleton. Zheng et al. [17] proposed a two-layer HITL optimization framework to numerically solve the matching condition and customize LLE assistance for individual users. This matching condition was used to drive an energy shaping law for the LLE, ultimately delivering enhanced gait performance. Despite these advancements, Huang et al. [14] raised concerns regarding the limitations in minimizing human energy costs through the quantification of metabolic costs. They introduced a reinforcement learning-based HITL optimization method, which optimized the switching time of gait stages via policy iterations to provide optimal mechanical energy for the hip exoskeleton. Gregg et al. [18] conducted an optimization search for a key parameter set of an optimal energy forming control algorithm. This set included proportionality coefficients related to gravity and inertia shaping, with the cost function being the square integral of the human joint torque. Subsequently, the optimal parameter set was identified by exploiting CMA-ES optimization in a simulated environment. Li et al. [19] extended the application of the HITL optimization by proposing an adaptive control strategy, aimed at enhancing the intuitiveness and usability of a soft exo-suit. The cost function was designed to minimize the tracking position/velocity errors. They further proposed a barrier energy function with respect to center of mass (COM) and zero moment point in HITL control [3].

The aforementioned studies primarily calculate the cost function for the HITL optimization based on metabolic cost [20]. However, some researchers suggest that human subjective judgment of preferences can also be integrated into the HITL optimization [21,22]. Tucker et al. [22] conceptualized the concept of dueling bandit problem to address the objective that cannot be quantitatively measured, and proposed the CoSpar HITL optimization algorithm. The CoSpar algorithm leverages human preferences as feedback to optimize gait trajectories. To handle the complexities of high-dimensional optimization driven by human preferences, the LineCoSpar algorithm was developed as an extension of CoSpar. Through HITL experiments on exoskeletons, the optimal set of gait parameters such as step length and step width were determined.

The investigation of optimizing HITL systems using feedback from metabolic costs [5,13,16,17,23,24,25], qualitative feedback [26], and walking speed [19] holds significant importance for the design and enhancement of LLEs [27,28,29]. However, there remains a gap in research on gait trajectory planning that simultaneously considers the dynamics of the HMS and human physiological responses. For instance, the muscle activity-based HITL optimization was proved to be faster than that based on metabolic cost [20]. This paper proposes a novel surface electromyography (sEMG)-based HITL motion planning method. This approach integrates offline trajectory optimization with online trajectory selection, sEMG signals are utilized as biofeedback and a hybrid dynamical model of the HMS is deduced and incorporated into the gait trajectory optimization process. The proposed method is validated on an LLE platform, demonstrating its effectiveness in optimizing gait trajectories to achieve stable assisted walking while prioritizing human biofeedback. The main contribution is threefold:

(1) A five-degree-of-freedom hybrid dynamical model of the HMS is derived. Among them, the continuous and discrete kinetics models are built using the Lagrangian method. Subsequently, the state space equation of the hybrid dynamical system is established, which encapsulates both the continuous and discrete dynamic processes, laying a foundation for gait trajectory optimization.

(2) The proposed method integrates offline trajectory optimization with online trajectory selection. Specifically, a direct collocation-based offline trajectory optimization method is proposed, converting the continuous trajectory optimization problem into a nonlinear program aimed at minimizing an energy-dependent objective function. This process results in an optimized gait library, which includes various gait trajectories that considers dynamic constraints and additional walking constraints in accordance with the principle of energy optimality.

(3) A sEMG-based HITL optimization method is proposed. The sEMG signals are utilized as human biofeedback to calculate the cost function through the Thompson sampling method. This enables the iterative selection of an optimal gait trajectory from the precomputed gait library, ensuring that the selected trajectory aligns with human physiological responses.

## 2. Hybrid Dynamics Modeling

**Figure 1:**
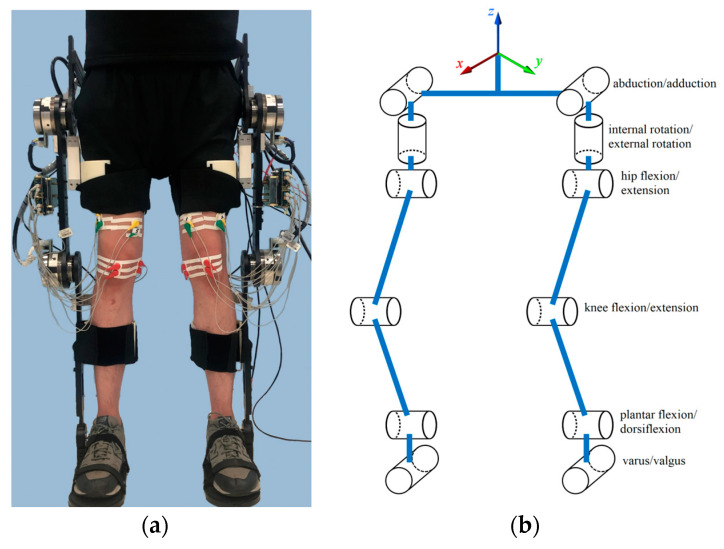
The HMS model. (**a**) The real HMS. (**b**) The ideal HMS model.

**Figure 2:**
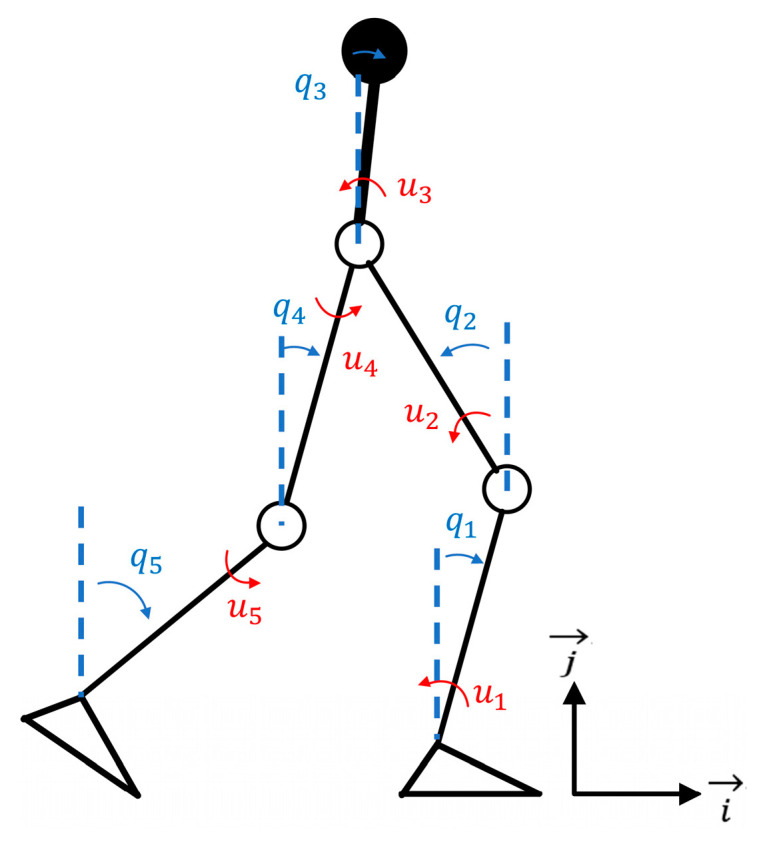
The simplified five-link hybrid HMS model.

The human–robot interaction system is modeled as a rigid body system, which is represented by a kinematic tree and shown in Figure 1. Both the human lower limb and LLE have 12 degrees of freedom (DOFs) [1]. To reveal the dynamical behavior of the HMS and avoid overcomplication of the dynamical model, the HMS is simplified as a five-link hybrid dynamical model depicted in Figure 2, the upper body of the human is modeled as a single rigid link and attached to the torso of the LLE. The human does not provide actuation and fully follows the LLE, the human masses and inertias are combined in the corresponding links of the LLE [6]. The five-link hybrid dynamical model is subject to three assumptions, which is shown as below:

**Assumption** **1.** *The human and LLE are rigidly connected, and the human lacks the ability to generate an active joint force. They can be considered as a unified whole. The whole HMS is composed of five links with four revolute joints.*

**Assumption** **2.** *The HMS is limited to movement in the sagittal plane, which has four joints with torque inputs, namely the hip and knee joints of both lower limbs, while the ankle joints have no torque input. The HMS can be modeled as point-legged robots.*

**Assumption** **3.** *The gait motion of the HMS is characterized by alternating phases of a continuous, single-support swing phase and an instantaneous, double-support impact phase [6]. The double-support impact phase encompasses two single-support swing phases before and after transition, and the process is triggered by the impact between the swinging leg and the ground. This process occurs instantaneously and complies with the conservation of angular momentum.*

The validity of the five-link hybrid model mainly lies in three aspects: (1) treating the stance leg and swing leg as an integrated system to analyze the dynamic coupling of legs, (2) considering the underactuated issue of due to the absence of torque input from the stance leg, and (3) taking into account the abrupt change in velocity when the swing leg contacts with the ground, thus making the HMS involve both continuous and discrete dynamics. Developing a state space equation for the HMS is essential for trajectory optimization and motion control. This section focuses on the dynamics modeling of the five-link hybrid HMS to establish the state space equation.

Based on the beforementioned assumptions, the dynamic equation is based on rigid body dynamics modeling. This paper focuses on the forward dynamics modeling for deriving the state space equation of the system, combining (A14)–(A18) in Appendix A, the equation of the dynamical model of the swing phase is achieved as

(1) $$M\cdot \ddot{q}=\Gamma +\tau$$

where $\tau ={\left[\begin{array}{ccccc} \tau_{1} & \tau_{2} & \tau_{3} & \tau_{4} & \tau_{5} \end{array}\right]}^{T}$, $\ddot{q}$ is the generalized angular acceleration, M is an inertia matrix, Γ is a superposition of a Coriolis and centrifugal force term C and a gravitational term G independent of the generalized acceleration and generalized torque. M, C, and G are deduced based on the Lagrange equation, note that the mechanical parameters of the LLE have been presented in our previous work [30], and the mass and inertia of human are in proportion integrated into the respective links of the LLE. Human–machine integration is considered in dynamical modeling, so as to simplify the model and make it more applicable to the human who is unable to generate an active joint force of lower limb. Among them, Γ is shown as

(2) $$\Gamma =-C\cdot \dot{q}-G$$

Then, the dynamical model of the continuous swing phase can be transformed into a state space equation, which is addressed as

(3) $$x=\left[\begin{array}{c} q\\ \dot{q} \end{array}\right],\dot{x}=\left[\begin{array}{c} \dot{q}\\ \ddot{q} \end{array}\right]=f(x)+g(x)u=\left[\begin{array}{c} \mathrm{col}(x_{1},x_{2},x_{3},x_{4},x_{5})\\ M{\left(x\right)}^{-1}\cdot \Gamma (x) \end{array}\right]+B\cdot u$$

where $\dot{x}$ denotes state variables, including generalized velocity and acceleration, f,g are from the continuous-time and swing-phase dynamics of the dynamic equation of LLE, B is the input torque mapping matrix, and ***u*** is the input driving torque. B and ***u*** are defined as

(4) $$B=\left[\begin{array}{ccccc} 0 & 0 & 0 & 0 & 0\\ 0 & 0 & 0 & 0 & 0\\ 0 & 0 & 0 & 0 & 0\\ 0 & 0 & 0 & 0 & 0\\ 0 & 0 & 0 & 0 & 0\\ 1 & -1 & 0 & 0 & 0\\ 0 & 1 & -1 & 0 & 0\\ 0 & 0 & 1 & -1 & 0\\ 0 & 0 & 0 & 1 & -1\\ 0 & 0 & 0 & 0 & 1 \end{array}\right],\;u=\left[\begin{array}{c} u_{1}\\ u_{2}\\ u_{3}\\ u_{4}\\ u_{5} \end{array}\right]$$

Based on Assumption 3, the double-support phase can be modeled as a transient impact event between the swing foot and ground, with this phase being instantly completed. Under the assumption of rigid ground, the instantaneous impact process can be considered to have a coefficient of restitution equal to zero, leading to abrupt changes in state variables of the dynamic system. Mathematically, this process is characterized by the state variables encountering a switching surface *S* and being triggered by an impact map Δ(x). Subsequently, this map projects the state variables into a new state space. The impact map involves two main steps: first, during the transient impact, the position coordinates of the HMS remain unchanged, while the generalized velocities undergo a sudden change due to an instantaneous shift in generalized momentum [6]; second, the two single-support swing legs interchange their roles instantaneously within this phase.

We assume that on the *S*, before the impact mapping, the generalized coordinate is $q^{-}$ and the generalized velocity is ${\dot{q}}^{-}$. After the impact mapping, the generalized coordinate is $q^{+}$ and the generalized velocity is ${\dot{q}}^{+}$. Since there is mutual exchange between the stance leg and swing leg on the generalized coordinates at the impact event, the preimpact $q^{-}$ and the postimpact $q^{+}$ satisfy

(5) $$q^{+}=\left[\begin{array}{ccccc} 0 & 0 & 0 & 0 & 1\\ 0 & 0 & 0 & 1 & 0\\ 0 & 0 & 1 & 0 & 0\\ 0 & 1 & 0 & 0 & 0\\ 1 & 0 & 0 & 0 & 0 \end{array}\right]\cdot q^{-}$$

The mapping matrix in (5) swaps the generalized coordinates corresponding to each leg, without altering their actual numerical values, and this mapping matrix preserves the continuity of joint positions across the impact event, ensuring smooth transitions between preimpact and postimpact states. Equation (5) describes the process of just swapping the relevant generalized coordinates between the legs without modifying the position coordinates. Thus, the continuity of the joint positions is inherently preserved. The velocity variation between ${\dot{q}}^{-}$ and ${\dot{q}}^{+}$ can be calculated from (6) to (10) in accordance with the conservation of angular momentum because of impact event. The angular momentum of the swing leg $P_{5}^{-}$ is

(6) $$\hat{k}\cdot \sum_{i=1}^{5}\left((P_{ci}^{-}-P_{5}^{-})\times (m_{i}{\dot{P}}_{ci}^{-})+{\dot{q}}_{i}^{-}I_{i}\cdot \hat{k}\right)=\hat{k}\cdot \sum_{i=1}^{5}\left((P_{ci}^{+}-P_{0}^{+})\times (m_{i}{\dot{P}}_{ci}^{+})+{\dot{q}}_{i}^{+}I_{i}\cdot \hat{k}\right)$$

The angular momentum of the knee joint of the swing leg $P_{4}^{-}$ is

(7) $$\hat{k}\cdot \sum_{i=1}^{4}\left((P_{ci}^{-}-P_{4}^{-})\times (m_{i}{\dot{P}}_{ci}^{-})+{\dot{q}}_{i}^{-}I_{i}\cdot \hat{k}\right)=\hat{k}\cdot \sum_{i=2}^{5}\left((P_{ci}^{+}-P_{1}^{+})\times (m_{i}{\dot{P}}_{ci}^{+})+{\dot{q}}_{i}^{+}I_{i}\cdot \hat{k}\right)$$

The angular momentum of the hip joint of the swing leg $P_{3}^{-}$ is

(8) $$\hat{k}\cdot \sum_{i=1}^{3}\left((P_{ci}^{-}-P_{3}^{-})\times (m_{i}{\dot{P}}_{ci}^{-})+{\dot{q}}_{i}^{-}I_{i}\cdot \hat{k}\right)=\hat{k}\cdot \sum_{i=3}^{5}\left((P_{ci}^{+}-P_{2}^{+})\times (m_{i}{\dot{P}}_{ci}^{+})+{\dot{q}}_{i}^{+}I_{i}\cdot \hat{k}\right)$$

The angular momentum of the hip joint of the stance leg $P_{2}^{-}$ is

(9) $$\hat{k}\cdot \sum_{i=1}^{2}\left((P_{ci}^{-}-P_{2}^{-})\times (m_{i}{\dot{P}}_{ci}^{-})+{\dot{q}}_{i}^{-}I_{i}\cdot \hat{k}\right)=\hat{k}\cdot \sum_{i=4}^{5}\left((P_{ci}^{+}-P_{2}^{+})\times (m_{i}{\dot{P}}_{ci}^{+})+{\dot{q}}_{i}^{+}I_{i}\cdot \hat{k}\right)$$

The angular momentum of the knee joint of the stance leg $P_{1}^{-}$ is

(10) $$\hat{k}\cdot ((P_{c1}^{-}-P_{1}^{-})\times (m_{1}{\dot{P}}_{c1}^{-})+{\dot{q}}_{1}^{-}I_{1}\cdot \hat{k})=\hat{k}\cdot \left((P_{c5}^{+}-P_{4}^{+})\times (m_{i}{\dot{P}}_{c5}^{+})+{\dot{q}}_{5}^{+}I_{5}\cdot \hat{k}\right)$$

The system state of preimpact and postimpact is defined

(11) $$x^{-}=\left[\begin{array}{c} q^{-}\\ {\dot{q}}^{-} \end{array}\right],\;x^{+}=\left[\begin{array}{c} q^{+}\\ {\dot{q}}^{+} \end{array}\right]$$

where $x^{-}$ and $x^{+}$ represent the instantaneous preimpact state and postimpact state, respectively.

Combine (6)–(11), the discrete dynamic equation is expressed as

(12) $$x^{+}=\Delta (x^{-})$$

In all, the state space equation for the five-link hybrid HMS is formulated as

(13) $$\Sigma :\{\begin{array}{l} \begin{array}{ll} \dot{x}=f(x)+g(x)u, & x\notin S\\ x^{+}=\Delta (x^{-}), & x\in S \end{array} \end{array}$$

## 3. Human-in-the-Loop Motion Planning

**Figure 3:**
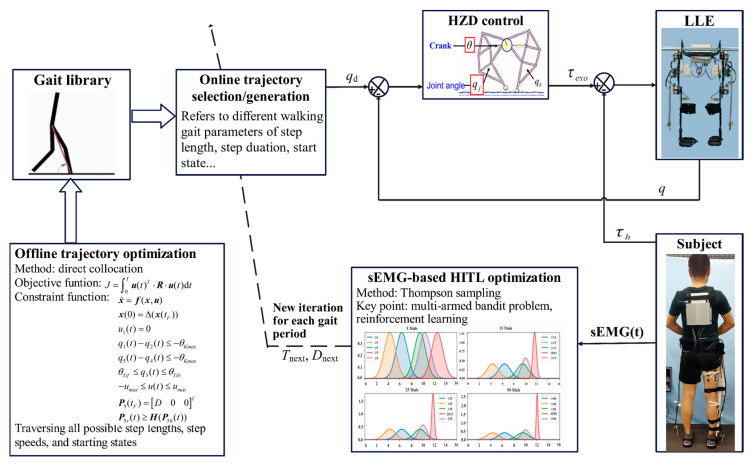
The proposed HITL motion planning method.

Gait trajectory planning affects the accuracy, stability, and comfort of the LLEs [31]. In order to solve the issue of insufficient control drive in HITL motion planning and find the optimal gait trajectory for wearers, we propose a method that integrates offline trajectory optimization with online trajectory generation. The offline trajectory optimization utilizes a direct collocation method based on the derived state space equation of the HMS. This method is employed to solve for the stability of the gait trajectory. The HITL optimization is based on Thompson sampling to identify the optimal gait trajectory for the wearers. The proposed HITL motion planning method is illustrated in Figure 3. The online gait trajectory planning relies on the gait library obtained from the offline trajectory optimization. sEMG signals are collected from the subject and used as human feedback for determining the cost function of the HITL optimization algorithm. The gait parameters of gait trajectory are updated by Thompson sampling, and the next gait trajectory is selected to perform the next iteration until an optimal value is found. Considering that the LLE is an underactuation system due to the ankle joints are solely controlled by passive components without driving torque input, and time-varying virtual constraint control method is sufficient and necessary for time-varying hybrid HMS to realizing stable walking [32]. Then, hybrid zero dynamics (HZD) control strategy [6,33] is applied to drive the LLE and assist walking according to the expected gait trajectory. The HZD controller is based on the mixed zero dynamics theory, appropriate virtual constraints are designed to ensure stable motion of the system. Based on the generated desired gait trajectory, the HZD controller compares the desired generalized coordinate $q_{d}$ with the actual generalized coordinate q obtained from the LLE and output the control torque $\tau_{\mathrm{exo}}$ by referring to the state space equation of HMS, The output $\tau_{\mathrm{exo}}$ drives the LLE in alignment with the desired trajectory. The human–exoskeleton interaction torque $\tau_{h}$ is measured by interaction force sensors mounted on links, which has a positive correlation with sEMG signals, and can be regarded as an external disturbance in the control system. This measurement can help achieve a better trajectory tracking control accuracy by adding it as a feedforward term in the motor current control loop. $\tau_{h}$ is treated as disturbances and integrated into the control process to dynamically adjust the generated torque $\tau_{\mathrm{exo}}$, ensuring that the movement of the LLE is closely aligned with human motion intention. This method enhances the robustness and adaptability of the LLE, making the system more responsive to the feedback of human.

This paper primarily focuses on the human-in-the-loop (HITL) motion planning. In this section, we provide a detailed explanation of both the offline trajectory and the HITL trajectory optimization processes.

### 3.1. Offline Trajectory Optimization

The direct collocation method is adopted for offline trajectory optimization, which transforms continuous trajectory optimization into nonlinear programming with finite multiple decision variables. The transformation process leverages multiple configuration points for polynomial interpolation approximation, thereby converting the differential equations into algebraic equations based on the obtained decision variables. Subsequently, the optimal trajectory can be searched out through nonlinear programming. To meet the requirements of high-dimensional planning with multiple constraints, low computational effort, and excellent convergence capability for gait trajectory planning on the LLEs, direct collocation proves to be a faster and more efficient way to address these needs [34].

The trajectory optimization issue is generally described by objective function and constraint function. According to (13), the hybrid dynamical HMS involves the optimization of numerous continuous-segment trajectories. Due to gait periodicity and leg symmetry during walking, the dynamical system can be restricted in single-swing phase, thus the trajectory optimization is simplified to single-segment continuous trajectory optimization. The gait trajectory optimization of the hybrid HMS is divided into objective function and constraint function. Based on the principle of energy optimality in the walking process, the objective function is defined as

(14) $$J=\int_{0}^{T}u{\left(t\right)}^{T}\cdot R\cdot u(t)dt$$

where J is the cost value, R is a quadratic matrix that belongs to a positive-definite matrix. If the quadratic matrix is a semi positive-definite matrix, it will result in input torque maintaining the upper limit value in the optimization result, some joints may exhibit behavior similar to bang-bang control. But a positive-definite matrix benefits the curves of input torque and state variables to be smooth, thereby the error of approximating the state trajectory can be reduced by using such smooth curves.

For the constraint functions, the hybrid dynamics equation is the primary constraint function, determining the feasibility of the gait trajectory for walking. Additionally, constraints of swing leg ground clearance, impact map, physiology limit in walking, and gait parameters are incorporated to restraint the lower-limb motions adaptive to human physical characteristics. Finally, a gait library containing various gait trajectories is established. Based on the objective function and constraint functions, the complete trajectory optimization problem can be described as

(15) $$\begin{array}{ll} \min_{t_{0},\;t_{F},\;x(t),\;u(t)} & J=\int_{0}^{T}u{\left(t\right)}^{T}\cdot R\cdot u(t)dt\\ s.t.\; & \dot{x}=f(x,u)\\ & x(0)=\Delta (x(t_{F}))\\ & u_{1}(t)=0\\ & q_{1}(t)-q_{2}(t)\leq -\theta_{Kmax}\\ & q_{5}(t)-q_{4}(t)\leq -\theta_{Kmax}\\ & \theta_{Tif}\leq q_{3}(t)\leq \theta_{Tib}\\ & -u_{max}\leq u(t)\leq u_{max}\\ & P_{5}(t_{F})={\left[D\;0\;0\right]}^{T}\\ & P_{5v}(t)\geq H(P_{5h}(t)) \end{array}$$

where $t_{F}$ is the time when the swing leg contacts with the ground, $u_{1}$ is the input torque of ankle joint, $\theta_{Kmax}$ is the protection margin of minor hyperextension of knee joint, $\theta_{Tib}$ is the maximum inclination angle of upper torso, $\theta_{Tif}$ is the maximum anteversion angle of upper torso, $u_{max}$ is the maximum output torque of four joints, $P_{5}(t_{F})$ is the position at the end of gait cycle of the swing leg along the generalized coordinates, *D* is the expected single-step length, *H* is the ground clearance constraint function, and $P_{5v}$ and $P_{5h}$ are, respectively, the vertical and horizonal component of terminal position of the swing leg $P_{5}$. The objective function is an energy optimality function. The constraint functions encompass dynamics equation constraint, impact map constraint, underactuation constraint, prohibition on excessive extension constraint of the knee joint of the stance leg, prohibition on excessive extension constraint of knee joint of the swing leg, upper body inclination angle constraint, upper limit constraint of the output torque, single-step length constraint, and ground clearance constraint for the swing leg, respectively.

Note that the decision variables in (15) contain infinite dimensional continuous vector functions, and it is unsolvable. The direct collocation method approximates the continuous objective function and constraint function through interpolation, which can be used to solve this problem, with introducing a discrete representation of the continuous time trajectory optimization. Correspondingly, the decision variables are transformed into the state and control variables of the collocation points on the trajectory. This transformation converts the original trajectory optimization problem into a nonlinear optimization problem that can be solved using modern optimization tools. As stated in (15), the states and control variables are approximated as discrete points. The target optimization trajectory is divided into *N* interpolation segments, each segment consists of three sampling points: the starting point, midpoint, and endpoint. The endpoint of one segment serves as the starting point for the next. The sampling points that connect interpolation segments are called nodes, and the sampling points that only exist in one interpolation segment are called midpoints. Both midpoints and nodes are called configuration points, the interpolation function of the dynamic equation is equal to its real function at these configuration points. This interpolation way benefits the improvement of approximation accuracy, helps maintain trajectory smoothness and mitigates the impact of state discontinuities between segments [6]. Consequently, there are 2*N* + 1 time sampling points on the target trajectory as shown in (16).

The duration of each interpolation segment is defined as $h_{k}=t_{k+1}-t_{k}$. Then, the states and control variables are also divided into 2*N* + 1 points listed in (17).

(16) $$t_{0},\cdots ,t_{k},t_{k+\frac{1}{2}},t_{k+1},\cdots ,t_{N}$$

(17) $$\begin{array}{c} x_{0},\cdots ,x_{k},x_{k+\frac{1}{2}},x_{k+1},\cdots ,x_{N}\\ u_{0},\cdots ,u_{k},u_{k+\frac{1}{2}},u_{k+1},\cdots ,u_{N} \end{array}$$

The quadratic interpolation function is utilized to approximate these sampling points, in accordance with the Newton–Cotes formula, the integral of the quadratic interpolation function r(t) within the interval [0,h] is calculated as

(18) $$\begin{array}{ll} W & =\int_{0}^{h}a+bt+ct^{2}dt\\ & =ah+\frac{1}{2}bh^{2}+\frac{1}{3}ch^{3}\\ & =\frac{h}{6}\left(r(0)+4r(\frac{h}{2})+r(h)\right) \end{array}$$

Referring to (18), the integral of one segment is expressed as

(19) $$x_{k+1}-x_{k}=\frac{1}{6}h_{k}\left(f(x_{k},u_{k})+4f(x_{k+\frac{1}{2}},u_{k+\frac{1}{2}})+f(x_{k+1},u_{k+1})\right)$$

where f is the hybrid dynamics equation and $x_{k}$ is the system state variable of the *k*-th node. $x_{k+\frac{1}{2}}$ represents the state of the system at the midpoint between two discrete time points $t_{k}$ and $t_{k+1}$, which can be deduced based on the application of Simpson’s rule for numerical integration, combined with Taylor series expansion. Thus, $x_{k+\frac{1}{2}}$ fulfills the constraint relationship with the starting point and endpoint [35] as

(20) $$x_{k+\frac{1}{2}}=\frac{1}{2}(x_{k}+x_{k+1})+\frac{h_{k}}{8}\left(f(x_{k},u_{k})-f(x_{k+1},u_{k+1})\right)$$

Equations (19) and (20) represent the system dynamics constraints based on the discrete decision variables, the middle state $x_{k+\frac{1}{2}}$ is consistent with the states at the surrounding nodes, thus enhancing the fidelity of the numerical solution while maintaining the continuity and smoothness of the trajectory.

Define the integrand as $w(t)=u{\left(t\right)}^{T}\cdot R\cdot u(t)$, in a similar way of (18), thus the integral of the entire gait cycle can be expressed as

(21) $$\int_{t_{0}}^{t_{F}}w(t)dt\approx \sum_{k=0}^{N-1}\frac{h_{k}}{6}(w_{k}+4w_{k+\frac{1}{2}}+w_{k+1})$$

The target function is derived as

(22) $$J=\sum_{k=0}^{N-1}\frac{h_{k}}{6}(w_{k}+4w_{k+\frac{1}{2}}+w_{k+1})$$

Therefore, the original continuous trajectory optimization problem can be transformed into nonlinear programming as shown below:

(23) $$\begin{array}{ll} \min_{t_{0},t_{F},\;x_{1}\cdots x_{N},\;u_{1}\cdots u_{N}} & J=\sum_{k=0}^{N-1}\frac{h_{k}}{6}(w_{k}+4w_{k+\frac{1}{2}}+w_{k+1})\\ s.t. & x_{k+1}-x_{k}=\frac{h_{k}}{6}\left(f(x_{k},u_{k})+4f(x_{k+\frac{1}{2}},u_{k+\frac{1}{2}})+f(x_{k+1},u_{k+1})\right)\\ & x_{0}=\Delta (x_{F})\\ & u_{1,k}=0\\ & q_{1,k}-q_{2,k}\leq -\theta_{Kmax}\\ & q_{5,k}-q_{4,k}\leq -\theta_{Kmax}\\ & \theta_{Tif}\leq q_{3,k}\leq \theta_{Tib}\\ & -u_{max}\leq u_{2\sim 5,k}\leq u_{max}\\ & P_{5,k}={\left[\begin{array}{ccc} D & 0 & 0 \end{array}\right]}^{T}\\ & P_{5v,k}\geq H(P_{5h,k}) \end{array}$$

Equation (23) can be solved using the *fmincon* function. The optimized discrete states and control variables should be restored to the expected continuous trajectory by the same interpolation method. Finally, the local time variable within the interpolation segment can be set as

(24) $$\tau =t-t_{k}$$

Then, the interpolation trajectory is reconstructed as

(25) $$u(t)=\frac{2}{h_{k}^{2}}\cdot \left(u_{k}\cdot (\tau -h_{k})(\tau -\frac{h_{k}}{2})-2u_{k+\frac{1}{2}}\cdot \tau (\tau -h_{k})+u_{k+1}\cdot \tau (\tau -\frac{h_{k}}{2})\right)$$

The derivative of the state variables for the hybrid dynamics equation is reconstructed as

(26) $$\begin{array}{l} \dot{x}(t)=f(x(t),u(t)),\\ \dot{x}(t)=\frac{2}{h_{k}^{2}}\left(f(x_{k},u_{k})\cdot (\tau -\frac{h_{k}}{2})(\tau -h_{k})-f(x_{k+\frac{1}{2}},u_{k+\frac{1}{2}})\cdot 2\tau (\tau -h_{k})+f(x_{k+1},u_{k+1})\cdot \tau (\tau -\frac{h_{k}}{2})\right) \end{array}$$

And the state variables can be achieved via the integration of the dynamic equations and shown as

(27) $$\begin{array}{cc} x(t) & =\int_{0}^{t}\dot{x}(\tau )d\tau \\ & =x_{k}+\left(\begin{array}{l} f(x_{k},u_{k})\cdot \frac{\tau }{h_{k}}+\frac{1}{2}\left(-3f(x_{k},u_{k})+4f(x_{k+\frac{1}{2}},u_{k+\frac{1}{2}})-f(x_{k+1},u_{k+1})\right)\cdot {\left(\frac{\tau }{h_{k}}\right)}^{2}\\ +\frac{1}{3}\left(2f(x_{k},u_{k})-4f(x_{k+\frac{1}{2}},u_{k+\frac{1}{2}})+2f(x_{k+1},u_{k+1})\right)\cdot {\left(\frac{\tau }{h_{k}}\right)}^{3} \end{array}\right)\cdot h_{k} \end{array}$$

We list the gait parameters of the offline and fine-turning gait trajectories to be optimized in Table 1. *T* and *D* traverse all values within the specific range, resulting in multiple optimized gait trajectories that are then compiled into a gait library.

### 3.2. HITL Trajectory Optimization

The gait library obtained from offline trajectory optimization generates natural and energy-efficient walking trajectories, facilitating the HMS in realizing stable walking under HZD control. However, the gait trajectory that personalizes the specific wearer is not guaranteed. Therefore, it is essential to establish appropriate gait parameters to fit physiological response of HMS during walking. Typically, human physiological response is subjective, its cost function cannot be measured directly through quantitative metrics but can be analyzed through biofeedback. Hence, a sEMG-based HITL optimization method is proposed, where the sEMG signals are utilized as human biofeedback. The HITL optimization involves the process of identifying the optimal gait trajectory within the gait library that yields the minimum value of the cost function. Since the Thompson sampling algorithm can effectively balance exploration and utilization through parameter setting, as well as provide a probability distribution curve of the cost function value [36], this paper utilizes Thompson sampling for conducting the HITL optimization.

For the objective function of the Thompson sampling-based HITL optimization, referring to [37], sEMG signals are used as qualitative feedback to assess user effort of muscle activity in accordance with the natural energetic optimization law. An objective function based on sEMG signals is designed as

(28) $$J=\frac{1}{T}\int_{0}^{T}\overset{N}{\underset{i=1}{\sum }}s_{i}dt$$

where *J* is the maximum objective in optimization, *N* is the channels of collecting sEMG signals, $s_{i}$ represents the sEMG signals of the *i*-th channel, and *T* is the single-step duration. Assuming that there are trajectories and their corresponding objective function values follow a Gaussian distribution. The prior distribution of the objective function values for each gait trajectory is designed as

(29) $$J_{i}\sim N(u_{0},\sigma_{0}^{2})$$

where $J_{i}$ is the objective function value of the *i*-th alternative gait trajectory, and $u_{0}$ and $\sigma_{0}^{2}$ are the mean and variance of the prior probability distribution, respectively.

Then, the probability function corresponding to the objective function value of real gait trajectory is called the likelihood function, which is shown as

(30) $$\mathbb{P}(J_{i}|u_{i})=N(u_{i},{\sigma_{i}}^{2})$$

where $u_{i}$ and $\sigma_{i}^{2}$ are, respectively, the mean and variance of the likelihood function of the *i*-th alternative gait trajectory subject to Gaussian distribution.

The posterior and prior probability distributions are congruent to conjugate distributions, and then the posterior probability distribution can be computed as

(31) $$\mathbb{P}(u_{i}|J_{i}^{1},J_{i}^{2},\cdots ,J_{i}^{n})=N\left(\frac{\frac{\mu_{0}}{\sigma_{0}^{2}}+\frac{\sum_{i=1}^{n}x_{i}}{{\sigma_{i}}^{2}}}{\frac{1}{\sigma_{0}^{2}}+\frac{n}{{\sigma_{i}}^{2}}},{\left(\frac{1}{\sigma_{0}^{2}}+\frac{n}{{\sigma_{i}}^{2}}\right)}^{-1}\right)$$

where $J^{1},J^{2},\cdots ,J^{n}$ are the feedbacks in HITL. When $J_{i}$ returns a new value, the posterior probability is immediately updated. Thompson sampling is then conducted on the posterior probability distribution, and ultimately a selection decision is made based on the maximum sampled value through comparison with the sampled values of all alternative trajectories. This selection decision implies that the optimal gait trajectory for the wearer has been identified. The selection decision strategy is

(32) $$a_{t}=\arg\max_{k}{\hat{u}}_{k},\;u_{k}\sim \mathbb{P}(u_{k}|r_{i}^{1},r_{i}^{2},\cdots ,r_{i}^{n})$$

where ${\hat{u}}_{k}$ is the sampling value of $u_{k}$, $a_{t}$ is the selection action at *t* moment.

## 4. Simulation and Experiment

Firstly, numerical simulation is performed to verify the derived hybrid dynamical model of the system and the HZD control method. The control simulation model is established using MATLAB/Simulink 2021: the dynamic mathematical model of the five-link hybrid system is the controlled object in Simulink, an online trajectory planning algorithm for a gait library that is generated by the direct collocation method, and an input–output feedback linearization algorithm based on time-varying virtual constraints are implemented in MATLAB language.

For the simulation parameters, a gait trajectory with *T* = 0.5 s and *D* = 0.4 m is set, the parameters of the controlled object keep the same as offline optimization, the control algorithm frequency is set to 500 Hz in consistency with actual deployment frequency, the coefficients of the PD controller with linearized input–output feedback are set to 10, and the human–exoskeleton interaction torque is considered as a disturbance with peak-to-peak value being 10 Nm, this disturbance is applied to the underactuated first joint. The output ***H*** matrix is defined as

(33) $$H=\left[\begin{array}{ccccc} 0 & 1 & 0 & 0 & 0\\ 0 & 0 & 1 & 0 & 0\\ 0 & 0 & 0 & 1 & 0\\ 0 & 0 & 0 & 0 & 1 \end{array}\right],\;H\cdot q=\left[\begin{array}{c} q_{2}\\ q_{3}\\ q_{4}\\ q_{5} \end{array}\right].$$

Then, to further validate the proposed sEMG-based HITL motion planning method on the LLE prototype, an LLE platform is established and a HITL optimization and control experiment is conducted.

**Figure 4:**
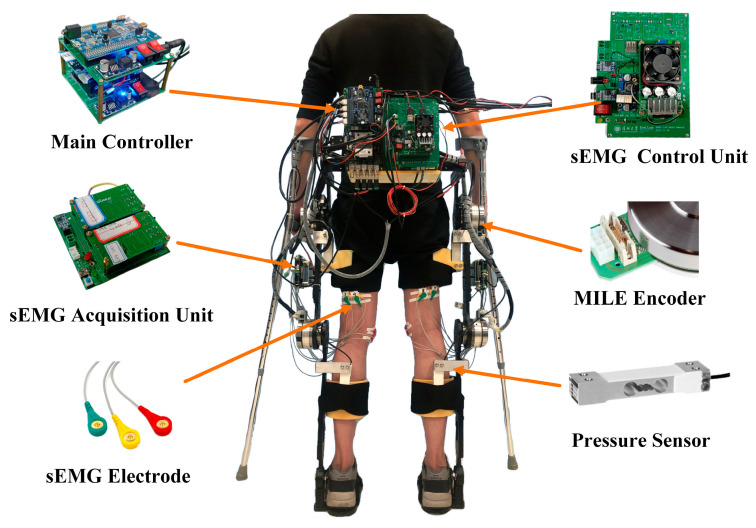
The experimental platform.

Figure 4 depicts the LLE prototype deployed with sensing and control networks. The primary control board model is F28377D, which supports embedded motion planning and control algorithms, as well as motor drive regulation. The MILE encoder, sEMG sensor, and pressure sensor are responsible for acquiring joint angle, sEMG signal, and interaction force, respectively. Communication is facilitated by CAN bus.

**Figure 5:**
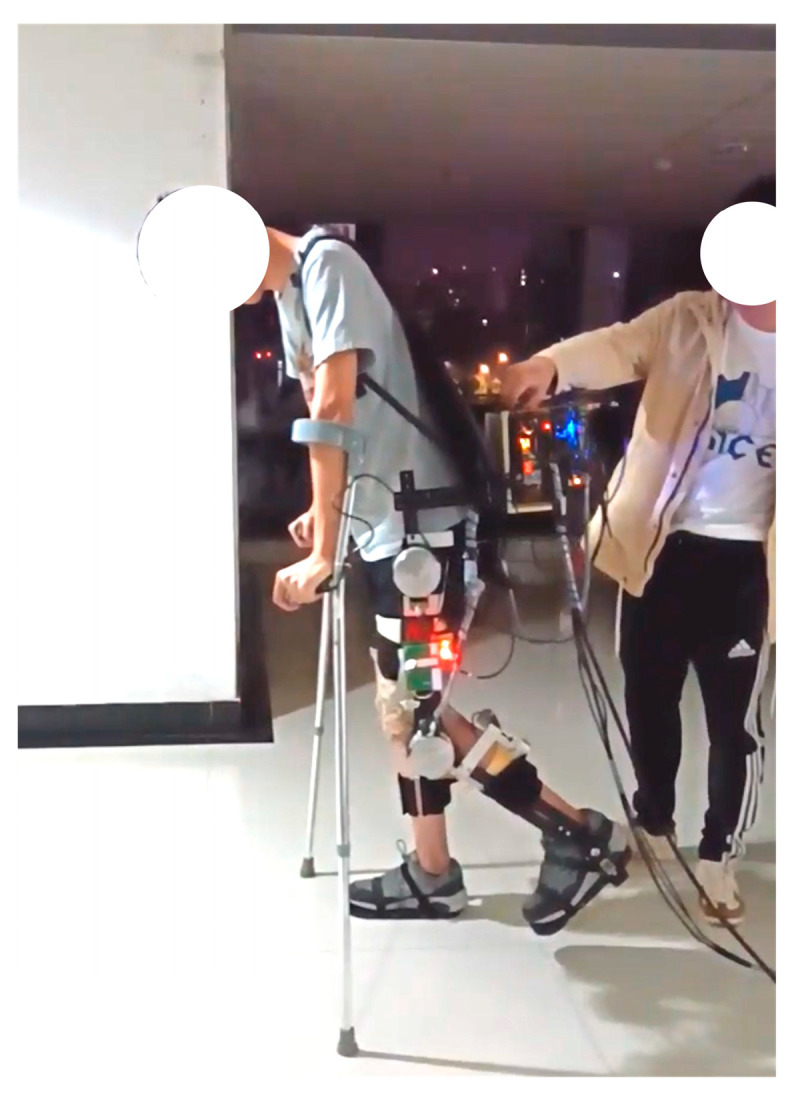
Assisted walking experiment with LLE.

Based on offline optimized gait trajectory for trajectory tracking control, a healthy subject wore LLE for assisted walking experiments, as shown in Figure 5. Eight lower-limb muscles were selected to attach sEMG electrodes for data collection, and the electrodes were secured with bandages to prevent displacement during walking. An emergency stop switch was installed on the cable, and an experimenter was assigned to monitor the total power supply of the LLE for safety. Additionally, the crutches used by the wearer were equipped with an emergency stop button to maintain balance when necessary. It does not provide driving force during walking. Based on the results of offline trajectory optimization, gait parameters from the gait library were selected to optimize LLE’s gait and implement the expected gait trajectory. The HZD control method was applied to achieve trajectory tracking of LLE. During walking, the subject was instructed to minimize the active force, and the driving torque of the LLE was the main force. Meanwhile, the joint angle, sEMG signal, and interaction force were measured by preplaced sensors.

In the HITL optimization control experiment, the collected sEMG signals of each gait cycle serve as human feedback for the Thompson sampling algorithm. The posterior probabilities are updated in accordance with the cost function. Thompson sampling is utilized to select the next gait trajectory from the gait library until convergence to the optimal trajectory. The experiment involves multiple periodic trajectories with different gait parameters. The estimation value of the variance for the Gaussian likelihood function is set to 1 for each gait trajectory. Additionally, 12 optimized transition gaits are introduced to enable natural switching between periodic gaits. The number of trials for the Thompson sampling-based HITL optimization is set to 50.

## 5. Result and Discussion

### 5.1. Result

#### 5.1.1. Simulation Result

**Figure 6:**
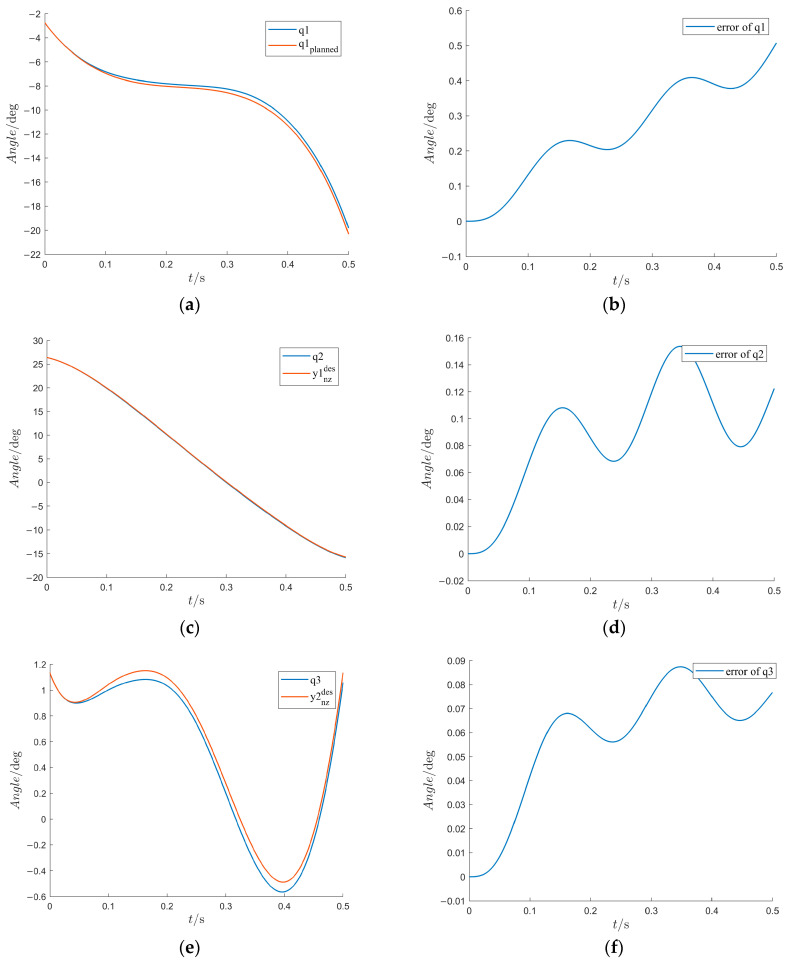

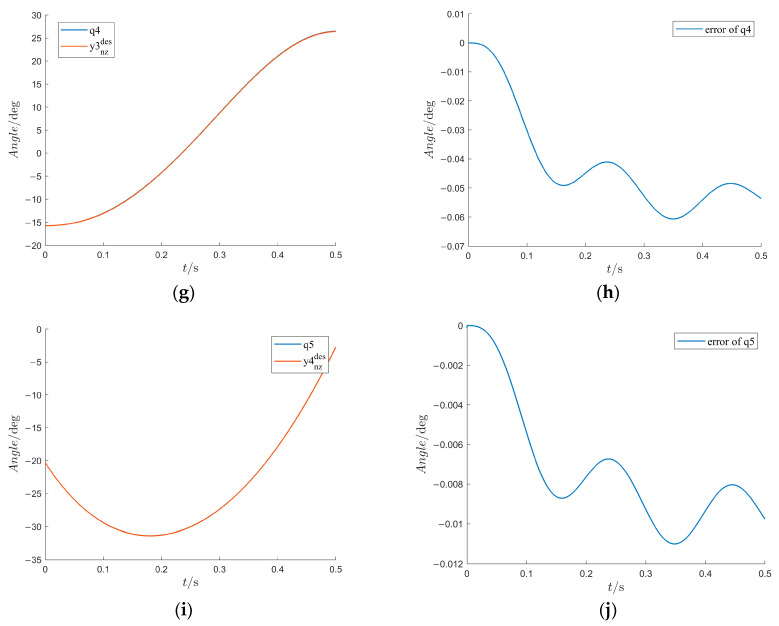
Trajectories and error curves of generalized coordinates under HZD control. (**a**) The virtual constraint control trajectory of the first generalized coordinate (the inclination angle of the stance leg shank). (**b**) The control error of the first generalized coordinate. (**c**) The virtual constraint control trajectory of the second generalized coordinate (the inclination angle of stance leg thigh). (**d**) The control error of the second generalized coordinate. (**e**) The virtual constraint control trajectory of the third generalized coordinate (the inclination angle of the upper torso). (**f**) The control error of the third generalized coordinate. (**g**) The virtual constraint control trajectory of the fourth generalized coordinate (the inclination angle of the swing leg thigh). (**h**) The control error of the fourth generalized coordinate. (**i**) The virtual constraint control trajectory of the fifth generalized coordinate (the inclination angle of the swing leg shank). (**j**) The control error of the fifth generalized coordinate.

Based on the simulation experiment, the trajectory tracking control results are displayed in Figure 6. We can see that under the influence of a disturbance torque, the tracking error of the first joint reaches its highest level of 0.5°; the tracking error of other actuated joints remains small though there is coupling interference from the disturbed first joint, with a maximum not exceeding 0.16°. However, the tracking error in the trajectory of the upper torso slightly increases, as shown in Figure 6e, because of the fact that the inclination angle of the upper body trunk does not change significantly during walking. The simulation allows for the verification of the derived hybrid dynamical model accurately represents the actual behavior of the system. By comparing the simulation results with the expected trajectories, the accuracy and validity of the model is assessed. The simulation result demonstrate that based on the five-link hybrid dynamical model, the combination of the HZD control algorithm with trajectory optimization can enable stable walking for the HMS that has underactuated and hybrid dynamic characteristics.

#### 5.1.2. Experimental Result

**Figure 7:**
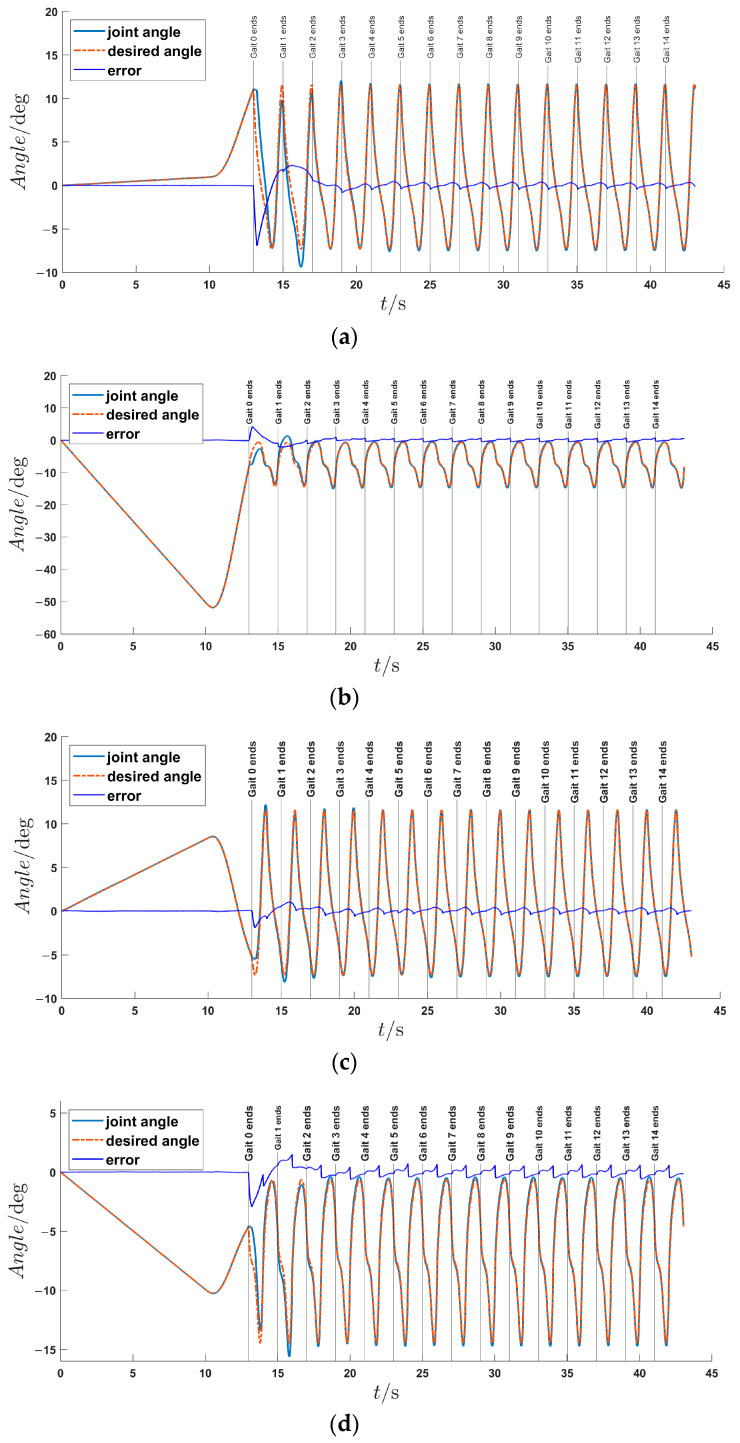
Trajectory tracking results during assisted walking. (**a**) Trajectory tracking curves and errors of the left hip joint. (**b**) Trajectory tracking curves and errors of the left knee joint. (**c**) Trajectory tracking curves and errors of the right hip joint. (**d**) Trajectory tracking curves and errors of the right knee joint.

Each optimized trajectory in the gait library is designated as the required trajectory for trajectory tracking control. An LLE-based assisted walking experiment is conducted with gait parameters of *T* = 1 s and *D* = 0.2 m. The trajectory tracking results of four joints are presented in Figure 7, with vertical lines marking the gait cycle number from Gait 0 to Gait 14. There is approximately 13 s of preparatory walking gait before Gait 0. It can be observed that the tracking error of each joint is less than 0.02° during the first 13 s of preparation gait due to the relatively low walking speed. Then, in the first two walking gaits, however, the tracking errors are somewhat large, with the maximum tracking errors not exceeding 6.9° for the left hip joint, 4.2° for the left knee joint, 1.9° for the right hip joint, and 2.9° for the right knee joint. At the end of Gait 4, convergence to a stable walking state is achieved asymptotically by LLE, with maximum tracking error of each joint being less than 0.8°. The root mean square (RMS) value of trajectory tracking is presented in Table 2. In total, neither the tracking error nor RMS value of each joint angle is less than 1.0° in stable walking state during gait trajectory tracking process.

**Figure 8:**
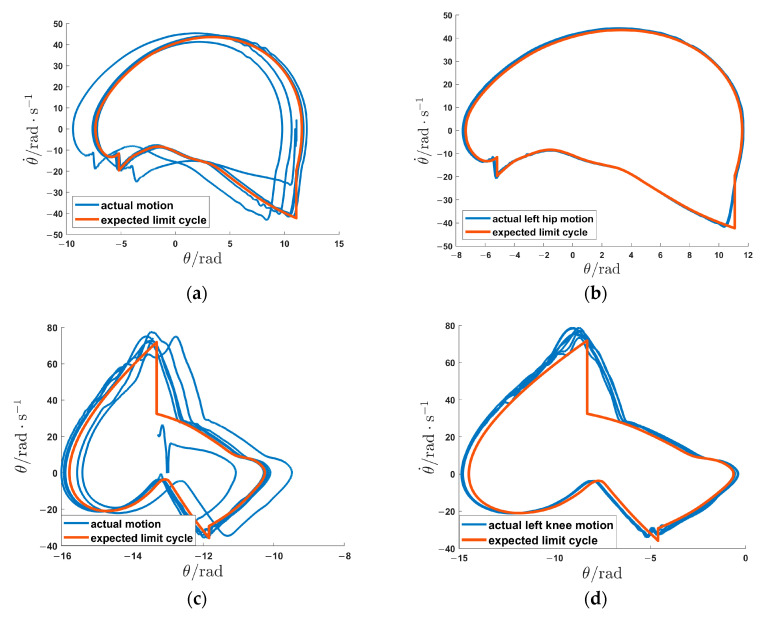

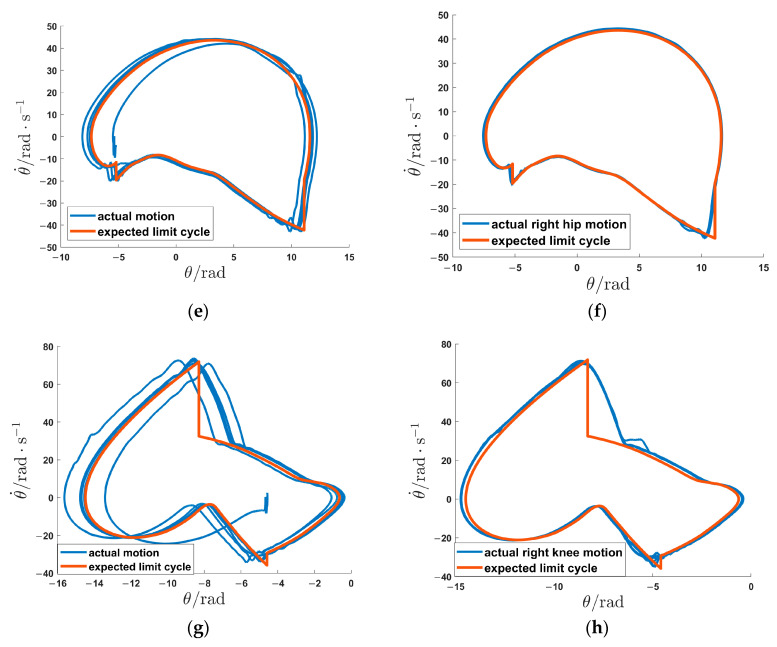
Phase portrait of walking process assisted by the LLE: (**a**,**c**,**e**,**g**) are the phase portraits of the left hip joint, left knee joint, right hip joint, and right knee joint in the early walking process (Gait 1 to Gait 6), respectively; (**b**,**d**,**f**,**h**) are the limit cycles of the left hip joint, left knee joint, right hip joint, and right knee joint in the mid-to-late walking process (Gait 7 to Gait 15), respectively.

To better evaluate the control efficacy based on the offline optimized gait trajectory, not only the tracking error, but also the phase portrait with respect to angle and angular acceleration are observed. The angles of each joint are Savitzky–Golay filtered [38], and numerical differentiation is performed to obtain the joint angular acceleration. Finally, the phase portrait of each joint in the walking process is drawn in Figure 8. From Figure 8a,c,e,g, during the early stages of walking, there is a noticeable disparity between the actual motion and the expected limit cycle. As for the mid-to-late stages of walking, Figure 8b,d,f,h indicate that the gait trajectory has undergone several gait cycles and gradually converges to the expected limit cycle. However, there are still some slightly larger errors in the discrete dynamic segments of the limit cycle, particularly for the left and right knee joints. This can be attributed to the limited bandwidth of the Savitzky–Golay filter, making it challenging to recover the mutational velocity signals.

From Figure 7 and Figure 8, it can be concluded that the offline optimization of gait trajectories via direct collocation is achievable and effective for natural and stable walking on the LLE, and the HMS achieves excellent trajectory tracking performance under the HZD control.

**Figure 9:**
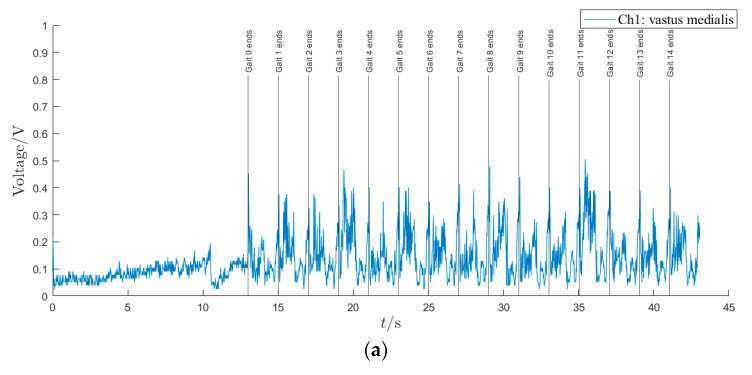

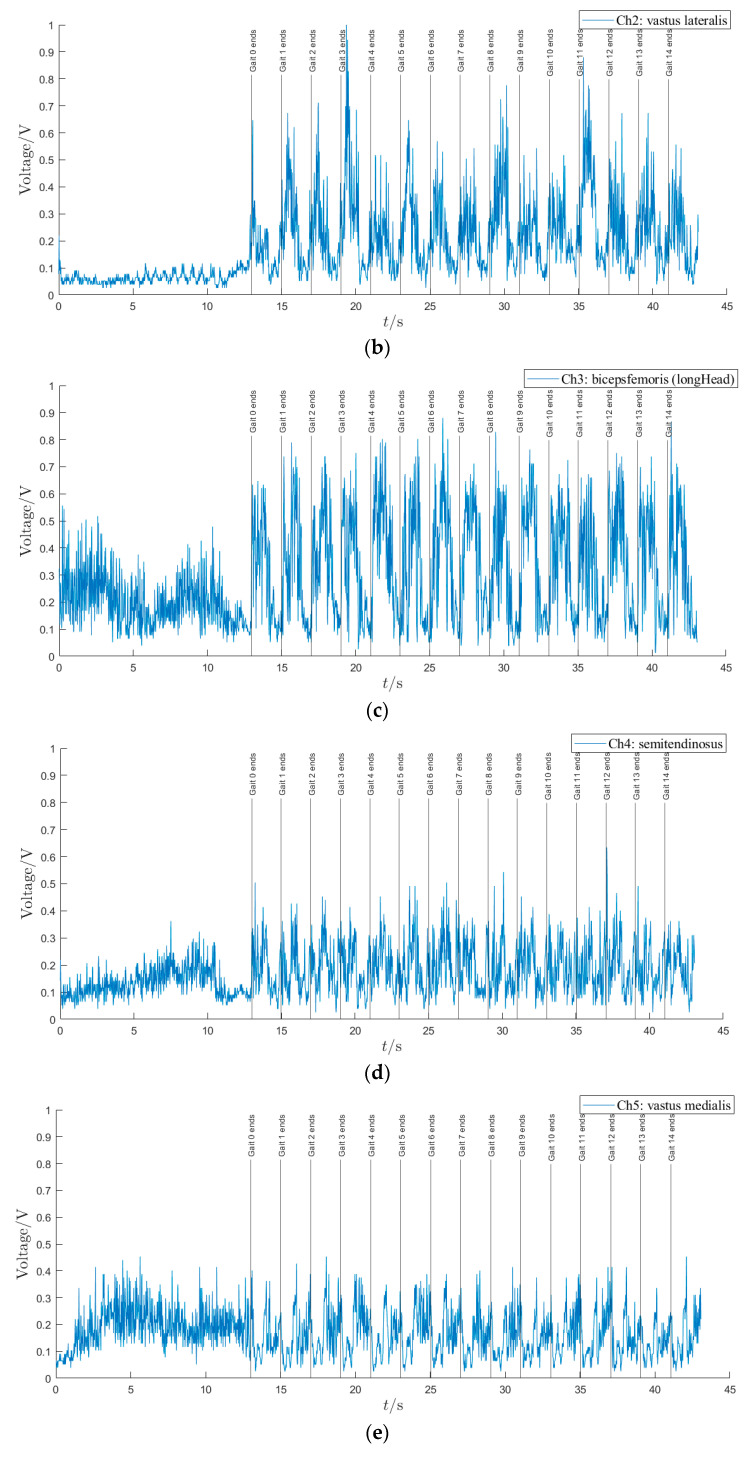

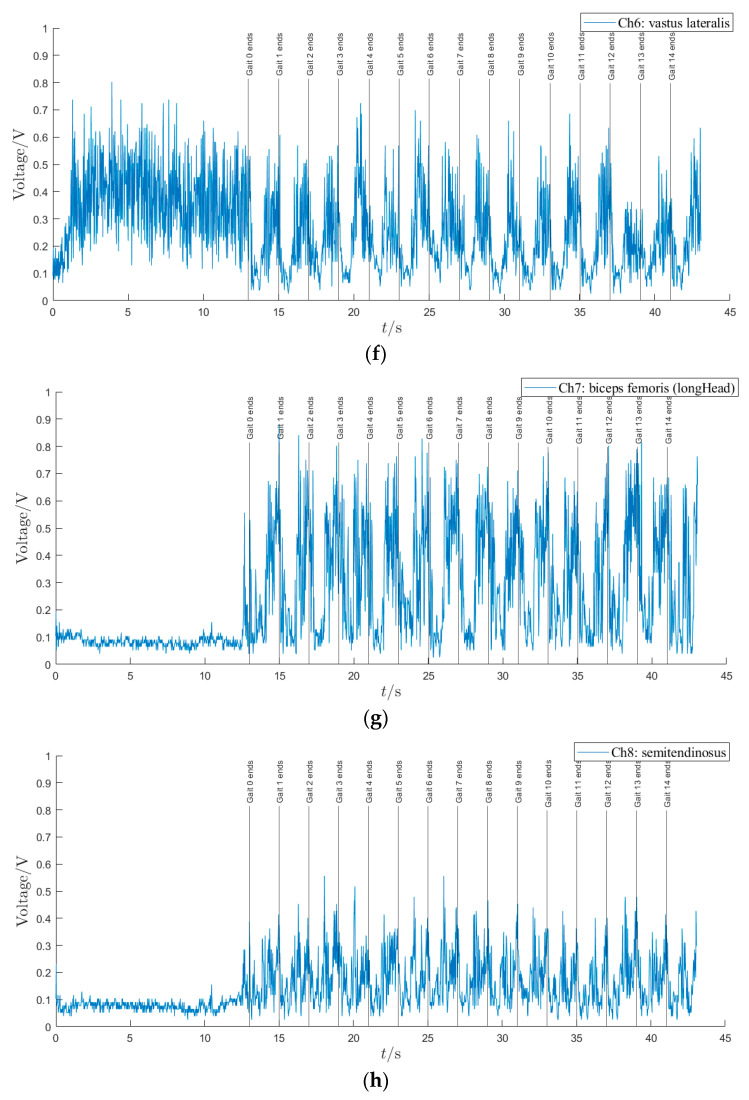
sEMG of eight channels during LLE assists walking. (**a**) The sEMG signals from vastus medialis of the left leg. (**b**) The sEMG signals from vastus lateralis of the left leg. (**c**) The sEMG signals from biceps femoris of the left leg. (**d**) The sEMG signals from semitendinosus of the left leg. (**e**) The sEMG signals from vastus medialis of the right leg. (**f**) The sEMG signals from vastus lateralis of the right leg. (**g**) The sEMG signals from biceps femoris of the right leg. (**h**) The sEMG signals from semitendinosus of the right leg.

Then, the sEMG signals from the vastus medialis, vastus lateralis, biceps femoris, and semitendinosus of both legs are collected and displayed in Figure 9. The intensity of the sEMG signals exhibits good periodicity, with a consistent trend of variability in each gait cycle. The sEMG signals from the muscles of the left and right legs demonstrate similarity during the periodic walk. Therefore, it is feasible to utilize sEMG signals as human feedback to calculate the cost function for the HITL optimization.

**Figure 10:**
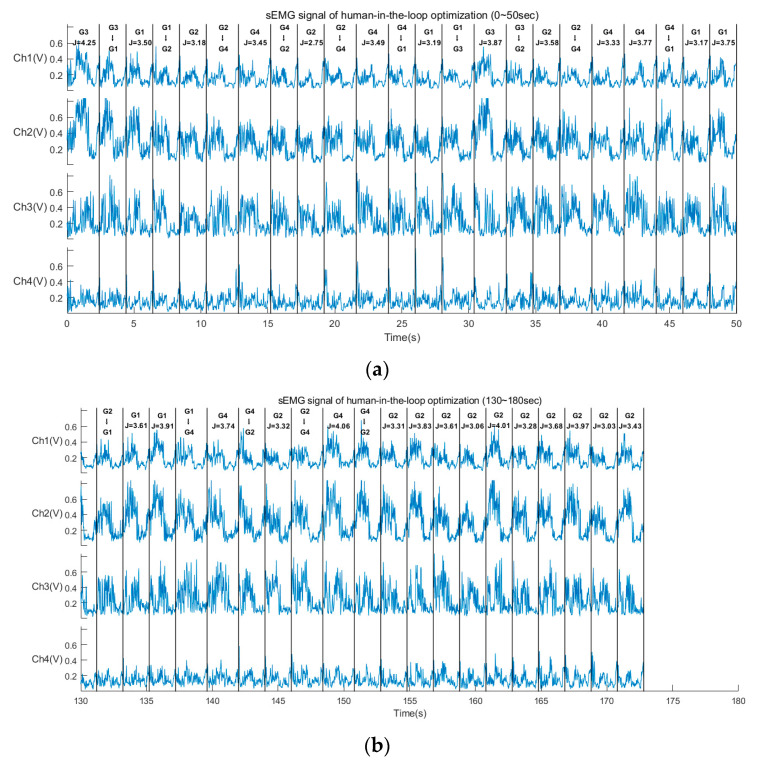
sEMG, value of cost function, and gait switching state in HITL. (**a**) Results at the first 50 s. (**b**) Results at the last 50 s.

**Figure 11:**
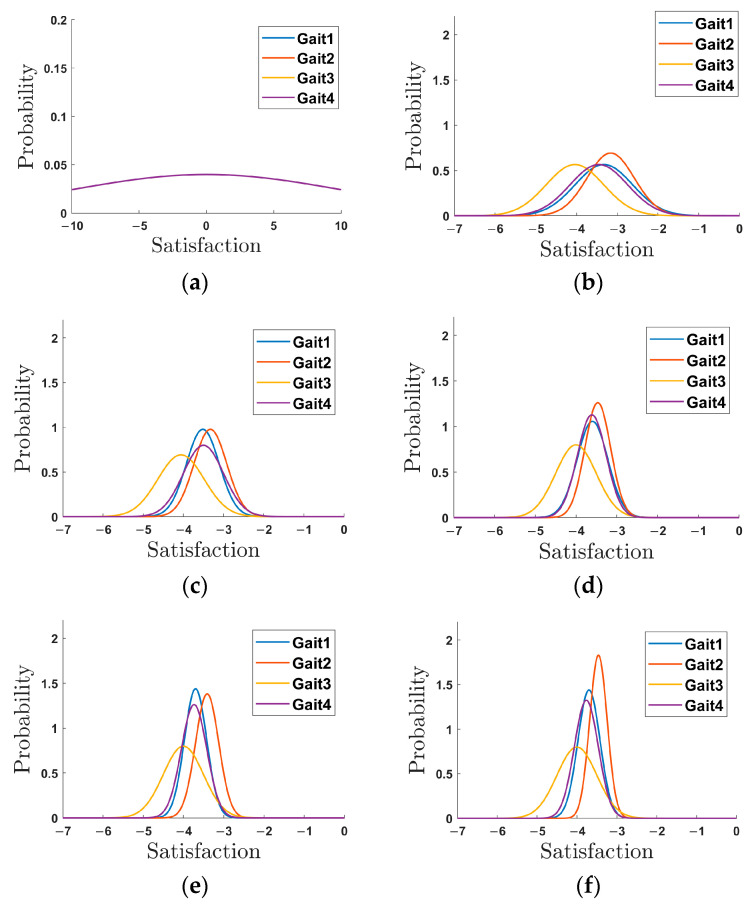
The curves of probability density function of each gait trajectory in the HITL optimization. (**a**) Prior probability distribution. (**b**) The 10th iteration. (**c**) The 20th iteration. (**d**) The 30th iteration. (**e**) The 40th iteration. (**f**) The 50th iteration.

Next, for the HITL optimization control experiments, we utilized the sEMG signals as biofeedback and four alternate walking trajectories labeled with gait names from the gait library (listed in Table 3). The obtained results include sEMG signal, value of the cost function, and the gait switching state. These results for the first 50 s and last 50 s of the walking experiments are truncated and presented in Figure 10. The long vertical lines of Figure 9 are used to divide the gait cycle, with comments of gait names and values of the cost function serving as feedbacks for HITL. Figure 10 illustrates that the gait trajectory of Gait 2 is ultimately selected in the HITL optimization from 150 s to 180 s, after which it continues walking without changing the gait trajectory. In fact, the entire optimization process is completed in approximately 150 s. Furthermore, these cost function values are constantly utilized to update the posterior probability density distribution, as depicted in Figure 11. The abscissa of the probability density distribution is a negative expression, since the negative value of the cost function is taken to be the satisfaction used in Thompson sampling. In Figure 11, the probability density distribution function of each gait becomes taller and thinner with increasing iteration numbers. After 50 iterations for the HITL optimization, the posterior probability density distribution function of Gait 2 (*T* = 1.0 s, *D* = 0.3 m) could achieve the minimum value of the cost function, Gait 2 is selected as the optimal trajectory in subsequent assisted walking. The posterior mean and variance are 3.4626 and 0.0476, respectively. In summary, the Thompson sampling-based HITL optimization method is capable of discerning the distribution of cost function values and selecting the optimal gait trajectory.

In conclusion, the sEMG biofeedback-based HITL optimization method using Thompson sampling takes less than 180 s to find the optimal gait trajectory for the wearer. The appropriate gait trajectory that minimizes the energy cost and the ability to walk stably for the subject is *T* = 1.2 s and *D* = 0.3 m. The proposed HITL optimization method is suitable for cases where there are fewer optional gaits, and its superiority has been demonstrated.

### 5.2. Discussion

The impact map typically introduces discontinuities in the state variables. Despite this, the impact map is incorporated into the HMS dynamics modeling in our approach, which explicitly addresses the discontinuities caused by impacts during the gait cycle. By integrating hybrid zero dynamics (HZD) control with direct collocation-based trajectory optimization, our method ensures smooth transitions and robust performance across impact events. As shown in Figure 8, the gait trajectory gradually converges to the desired limit cycle after several gait cycles, the phase transition becomes smoother. Some other approaches also studied phase transition, neural networks or blended control strategies were often used to recognize gait phases and manage transitions between control systems or dynamic models [39,40,41]. While these methods effectively ensure smooth transitions during phase changes, the discontinuities introduced by impact events within the gait cycle were not directly addressed. Lhoste et al. [42] calculated weight distribution as the ratio of vertical ground reaction forces (GRF) to facilitate transitions between left and right stance models. In contrast to methods that primarily focus on phase recognition or model transitions, our approach leverages the impact map to optimize the trajectory, thereby generating the reference trajectory. The use of impact map provides a unique advantage by directly addressing impact-induced discontinuities, by combining the impact map with advanced control and optimization techniques, our approach offers a comprehensive solution that enhances the stability and smoothness of gait control.

For the Thompson sampling-based HITL optimization method, we select the intensity of sEMG signals as human biofeedback in order to calculate the cost function. This decision is based on that, sEMG signals are easily captured when human muscles generate active forces, requiring less time for collection compared to using metabolic cost [27]. As shown in Figure 9, sEMG signals exhibit periodicity in each gait cycle, with weaker signals indicating weak muscle activity and lower force generation during walking. We believe that a state of less active force corresponds to a relatively comfortable condition for humans while wearing the LLE. Generally, sEMG signals are time-varying and containing significant intent information [43], enabling the use of sEMG signals in decoding gait-related events and parameters [44] as well as active torque [45]. There are numerous studies concerning sEMG-based interactive control for exoskeletons [46], such as Zhu et al. [47] designed a voluntary control strategy based on sEMG-driven musculoskeletal model for an exoskeleton robot, joint torque and quasi-stiffness were estimated and used to adjust the degree of exoskeleton assistance and transfer stiffness. Chen et al. [48] proposed an sEMG-based admittance control method for an LLE to adjust the auxiliary mode. While the encoding process is intricate and real-time encoding and control is tremendously challenging. Direct analysis of sEMG signals ensures real-time performance. Therefore, we have opted for sEMG as human feedback for HITL optimization purposes.

During the HITL optimization process, as shown in Figure 11, the variation trend of the posterior probability density distribution function indicates that when the probability curve appears squat, it signifies lower confidence in the distribution of cost function values and a larger variance in the posterior probability distribution. Conversely, a steep and thin probability curve suggests higher confidence in the distribution of cost function values and a smaller variance. The curves of the posterior probability density distribution function corresponding to different gait trajectories vary from squat to tall and thin. After 50 iterations, Gait 2 is selected as the optimal choice, which aligns with Figure 10b. The experimental results demonstrate that the entire optimization process can be completed in approximately 150 s. The proposed Thompson sampling-based method requires less time for the optimization process compared to traditional optimization methods, such as those based on metabolic cost [13] and particle swarm optimization (PSO) algorithms [27]. The optimization time required is comparable to that of the HITL optimization based on muscle activity using Bayesian and CMA-ES approaches [20]. Zhang et al. [13] optimized exoskeleton assistance during walking by minimizing human energy costs through the HITL optimization, effectively reducing metabolic energy consumption and enhancing exoskeleton performance. However, this study emphasized energy reduction rather than time efficiency during the optimization process. Han et al. [27] developed a muscle-activity-based cost function to optimize multi-gait ankle exoskeleton assistance for the HITL optimization, employing PSO to determine the optimal muscle weight combination on four lower leg muscles to compose the cost function with maximum differences, thereby improving the time efficiency of HIL optimization. Xu et al. [20] addressed the challenges of time efficiency in the HITL optimization aimed at reducing muscle activity during walking, particularly for users with limited endurance, and used Bayesian and CMA-ES methods to shorten the optimization process, the time efficiency was reduced but may compromise other aspects of optimization, such as precision or adaptability. Our approach combines Thompson sampling with sEMG signals for online trajectory optimization, primarily focusing on the optimization time and the selection of gait trajectories. The effectiveness of the proposed method for the HITL optimization has been demonstrated.

Indeed, the Thompson sampling method is susceptible to becoming stuck in a repetitive pattern once it identifies the optimal choice based on previous feedback. In view of this, we propose increasing the estimation variance value of the Gaussian likelihood function. Specifically, the estimated variance should be set several times larger than its actual value, thereby amplifying the variance of the Gaussian probability density function under homologous gait. This adjustment aims to flatten the posterior probability distribution during early iterations and create overlapping posterior probability density functions for each gait trajectory. As a result, the selection probabilities for each gait trajectory can be equalized at the initial stage. This Thompson sampling method can be extended to more alternative gait trajectories for the HITL optimization.

Overall, the proposed sEMG-based HITL motion planning method has been verified to be effective, demonstrating excellent trajectory tracking performance and requiring less time to find the optimal gait trajectory. In the future, it is recommended that more subjects wearing the LLE be included in trials, and that quantitative evaluation of subjective and objective feedback can be combined and considered in the cost function of the HITL optimization algorithm to achieve better personalized results.

## 6. Conclusions

This paper proposed a HITL motion planning method that combines offline trajectory optimization and online trajectory selection. sEMG signals were used as human biofeedback. Firstly, a five-DOF hybrid dynamical HMS model was constructed using the Lagrangian method. Next, the direct collocation method was employed for offline trajectory optimization based on the energy optimality law, resulting in the acquisition of a gait library. The Thompson sampling method was then utilized in the HITL optimization by leveraging the intensity of sEMG signals as the cost function to search for an optimal appropriate gait trajectory within the gait library. The proposed method was demonstrated in the HITL optimization and control experiment. The experimental results indicated that: (1) the optimized gait trajectories within the gait library under HZD control were realizable and obtained excellent tracking performance for the LLE; (2) selecting sEMG intensity as human feedback to calculate the cost function value was suitable and feasible, as it enabled the HITL optimization method based on sEMG biofeedback to identify an appropriate gait trajectory within 150 s; and (3) this approach could identify an optimal gait trajectory that not only enhances human performance but also ensures stable walking. As such, it represents a preliminary solution to personalized motion planning issues.

## Figures and Tables

**Table 1:** Offline gait parameters need to be optimized.

Parameter	Definition	Value
$\theta_{Tif}$	Maximum anteversion angle	−0.02(rad)
$\theta_{Tib}$	Maximum inclination angle	0.02(rad)
$\theta_{Kmax}$	Protection margin	0.02(rad)
H(d)	Ground clearance of swing foot	$H(d)=-0.024d^{2}+0.024(m)$
T	Single-step duration	1,1.1,1.2,…,1.5(s)
D	Single-step length	0.2,0.22,0.24,…,0.4(m)

**Table 2:** RMS of trajectory tracking.

	Left Hip Joint	Left Knee Joint	Right Hip Joint	Right Knee Joint
Overall RMS	0.9986°	0.7138°	0.3380°	0.4964°
RMS during stable walking	0.2046°	0.3195°	0.2087°	0.2716°

**Table 3:** Candidate gait trajectories.

Parameter	Gait 1	Gait 2	Gait 3	Gait 4
*T*	1 s	1 s	1.2 s	1.2 s
*D*	0.2 m	0.3 m	0.2 m	0.3 m

## Data Availability

Data are unavailable due to privacy and ethical restrictions.

## List of Abbreviations

LLE	lower-limb exoskeleton
HMS	human–machine system
HITL	human-in-the-loop
CMA-ES	covariance matrix adaptation evolution strategy
COM	center of mass
sEMG	surface electromyography
DOF	degree of freedom
HZD	hybrid zero dynamics
CAN	controller area network
RMS	root mean square
GRF	ground reaction forces
PSO	particle swarm optimization

## Appendix A. Derivation of the Kinematic Model and the Dynamical Model

In order to construct the state space equation for the five-link hybrid HMS, the first kinematic model and the second dynamical model should be deduced, they are the basis of the state space equation.

For kinematic modeling, the coordinate system is set between the floating base and revolute joints. Based on the space vector method, the kinematic model is defined via the generalized coordinates q as shown in (A1), with three space base vectors $\hat{i},\hat{j},\hat{k}$ expressed in (A2).

(A1) $$q=\left[\begin{array}{c} q_{1}\\ q_{2}\\ q_{3}\\ q_{4}\\ q_{5} \end{array}\right]$$

(A2) $$\hat{i}=\left[\begin{array}{c} 1\\ 0\\ 0 \end{array}\right],\;\hat{j}=\left[\begin{array}{c} 0\\ 1\\ 0 \end{array}\right],\;\hat{k}=\left[\begin{array}{c} 0\\ 0\\ 1 \end{array}\right]$$

where *q*_1_, *q*_2_, *q*_3_, *q*_4_, *q*_5_ are the absolute vertical inclination angles of the links ranging from one to five, with the right-hand direction along the vertical paper being the positive direction, as depicted in Figure 2. The inertial parameters of the five-link model are presented in Table A1.

**Table A1:** The inertial parameters of the five-link model.

Nomenclature	Description
*l_i_*	The length of the *i*-th link
*lc_i_*	The distance from the center of mass of the *i*-th link to its origin coordinate along $\hat{i}$
*m_i_*	The mass of the *i*-th link
*I_i_*	The rotational inertia of the *i*-th link with respect to its center of mass

Then, the location of COM of the stance leg and swing leg can be derived with respect to the predefined parameters. The location of COM of the stance leg shank $P_{c1}$ is derived as

(A3)
$$P_{c1}=\left(\begin{array}{c} l_{c1}\sin\left(q_{1}\right)-l_{1}\sin\left(q_{1}\right)\\ l_{1}\cos\left(q_{1}\right)-l_{c1}\cos\left(q_{1}\right)\\ 0 \end{array}\right)$$

The location of COM of the stance leg thigh $P_{c2}$ is

(A4)
$$P_{c2}=\left(\begin{array}{c} l_{c2}\sin\left(q_{2}\right)-l_{2}\sin\left(q_{2}\right)-l_{1}\sin\left(q_{1}\right)\\ l_{1}\cos\left(q_{1}\right)+l_{2}\cos\left(q_{2}\right)-l_{c2}\cos\left(q_{2}\right)\\ 0 \end{array}\right)$$

The location of COM of the upper torso $P_{c3}$ is

(A5)
$$P_{c3}=\left(\begin{array}{c} l_{c3}\sin\left(q_{3}\right)-l_{2}\sin\left(q_{2}\right)-l_{3}\sin\left(q_{3}\right)-l_{1}\sin\left(q_{1}\right)\\ l_{1}\cos\left(q_{1}\right)+l_{2}\cos\left(q_{2}\right)+l_{3}\cos\left(q_{3}\right)-l_{c3}\cos\left(q_{3}\right)\\ 0 \end{array}\right)$$

The location of COM of the swing leg thigh $P_{c4}$ is

(A6) $$P_{c4}=\left(\begin{array}{c} l_{c4}\sin\left(q_{4}\right)-l_{2}\sin\left(q_{2}\right)-l_{1}\sin\left(q_{1}\right)\\ l_{1}\cos\left(q_{1}\right)+l_{2}\cos\left(q_{2}\right)-l_{c4}\cos\left(q_{4}\right)\\ 0 \end{array}\right)$$

The location of COM of the swing leg shank $P_{c5}$ is

(A7) $$P_{c5}=\left(\begin{array}{c} l_{4}\sin\left(q_{4}\right)-l_{2}\sin\left(q_{2}\right)-l_{1}\sin\left(q_{1}\right)+l_{c5}\sin\left(q_{5}\right)\\ l_{1}\cos\left(q_{1}\right)+l_{2}\cos\left(q_{2}\right)-l_{4}\cos\left(q_{4}\right)-l_{c5}\cos\left(q_{5}\right)\\ 0 \end{array}\right)$$

Assuming that the state variable is

(A8) $$x=\left(\begin{array}{c} q\\ \dot{q} \end{array}\right)$$

where $\dot{q}$ is the generalized velocity. The space linear velocity $v_{ci}$ of the COM satisfies:

(A9) $$v_{ci}=\frac{\partial p_{ci}}{\partial x}\cdot \dot{x}$$

Then, the $v_{ci}$ of the *i*-th link can be calculated as

(A10)
$$\begin{array}{ll} v_{c1} & =\left(\begin{array}{c} -{\dot{q}}_{1}\left(l_{1}\cos\left(q_{1}\right)-l_{c1}\cos\left(q_{1}\right)\right)\\ -{\dot{q}}_{1}\left(l_{1}\sin\left(q_{1}\right)-l_{c1}\sin\left(q_{1}\right)\right)\\ 0 \end{array}\right)\\ v_{c2} & =\left(\begin{array}{c} -{\dot{q}}_{2}\left(l_{2}\cos\left(q_{2}\right)-l_{c2}\cos\left(q_{2}\right)\right)-l_{1}{\dot{q}}_{1}\cos\left(q_{1}\right)\\ -{\dot{q}}_{2}\left(l_{2}\sin\left(q_{2}\right)-l_{c2}\sin\left(q_{2}\right)\right)-l_{1}{\dot{q}}_{1}\sin\left(q_{1}\right)\\ 0 \end{array}\right)\\ v_{c3} & =\left(\begin{array}{c} -{\dot{q}}_{3}\left(l_{3}\cos\left(q_{3}\right)-l_{c3}\cos\left(q_{3}\right)\right)-l_{1}{\dot{q}}_{1}\cos\left(q_{1}\right)-l_{2}{\dot{q}}_{2}\cos\left(q_{2}\right)\\ -{\dot{q}}_{3}\left(l_{3}\sin\left(q_{3}\right)-l_{c3}\sin\left(q_{3}\right)\right)-l_{1}{\dot{q}}_{1}\sin\left(q_{1}\right)-l_{2}{\dot{q}}_{2}\sin\left(q_{2}\right)\\ 0 \end{array}\right)\\ v_{c4} & =\left(\begin{array}{c} l_{c4}{\dot{q}}_{4}\cos\left(q_{4}\right)-l_{2}{\dot{q}}_{2}\cos\left(q_{2}\right)-l_{1}{\dot{q}}_{1}\cos\left(q_{1}\right)\\ l_{c4}{\dot{q}}_{4}\sin\left(q_{4}\right)-l_{2}{\dot{q}}_{2}\sin\left(q_{2}\right)-l_{1}{\dot{q}}_{1}\sin\left(q_{1}\right)\\ 0 \end{array}\right)\\ v_{c5} & =\left(\begin{array}{c} l_{4}{\dot{q}}_{4}\cos\left(q_{4}\right)-l_{2}{\dot{q}}_{2}\cos\left(q_{2}\right)-l_{1}{\dot{q}}_{1}\cos\left(q_{1}\right)+l_{c5}{\dot{q}}_{5}\cos\left(q_{5}\right)\\ l_{4}{\dot{q}}_{4}\sin\left(q_{4}\right)-l_{2}{\dot{q}}_{2}\sin\left(q_{2}\right)-l_{1}{\dot{q}}_{1}\sin\left(q_{1}\right)+l_{c5}{\dot{q}}_{5}\sin\left(q_{5}\right)\\ 0 \end{array}\right) \end{array}$$

The space linear velocity of each link is formulated as in (A10). where $v_{c1},v_{c2},v_{c3},v_{c4},v_{c5}$ are the space linear velocities of the 1-st to 5-th links, respectively.

Then, the angular velocity of the COM corresponding to each link is expressed as

(A11) $$\omega =\left(\begin{array}{c} \omega_{1}\\ \omega_{2}\\ \omega_{3}\\ \omega_{4}\\ \omega_{5} \end{array}\right)=\left(\begin{array}{c} {\dot{q}}_{1}\\ {\dot{q}}_{2}\\ {\dot{q}}_{3}\\ {\dot{q}}_{4}\\ {\dot{q}}_{5} \end{array}\right)$$

where ω is the angular velocity vector, $\omega_{1},\omega_{2},\omega_{3},\omega_{4},\omega_{5}$ are the angular velocities of each link along with $\hat{k}$ axis.

The dynamical model can be derived in reference to the Lagrange scheme shown in (A12).

(A12) $$Q=\frac{d}{dt}\frac{\partial L}{\partial \dot{q}}-\frac{\partial L}{\partial q}$$

where Q is the generalized force, L is the Lagrangian. The input driving torque of each joint τ has the following relationship with respect to Q:

(A13) Q=B⋅τ⋅

where B is a torque mapping matrix. Referring to (A12) and (A13) and the deduced kinematic model, the driving torque of each joint in the swing phase can be derived as

(A14)
$$\begin{array}{cc} \tau_{1}= & I_{1}{\ddot{q}}_{1}+{l_{1}}^{2}m_{1}{\ddot{q}}_{1}+{l_{1}}^{2}m_{2}{\ddot{q}}_{1}+{l_{1}}^{2}m_{3}{\ddot{q}}_{1}+{l_{1}}^{2}m_{4}{\ddot{q}}_{1}+{l_{1}}^{2}m_{5}{\ddot{q}}_{1}+\\ & {l_{c1}}^{2}m_{1}{\ddot{q}}_{1}-2l_{1}l_{c1}m_{1}{\ddot{q}}_{1}-gl_{1}m_{1}\sin\left(q_{1}\right)-gl_{1}m_{2}\sin\left(q_{1}\right)\\ & -gl_{1}m_{3}\sin\left(q_{1}\right)-gl_{1}m_{4}\sin\left(q_{1}\right)-gl_{1}m_{5}\sin\left(q_{1}\right)\\ & +gl_{c1}m_{1}\sin\left(q_{1}\right)+l_{1}l_{2}m_{2}{\ddot{q}}_{2}\cos\left(q_{1}-q_{2}\right)+l_{1}l_{2}m_{3}{\ddot{q}}_{2}\cos\left(q_{1}-q_{2}\right)\\ & +l_{1}l_{2}m_{4}{\ddot{q}}_{2}\cos\left(q_{1}-q_{2}\right)+l_{1}l_{2}m_{5}{\ddot{q}}_{2}\cos\left(q_{1}-q_{2}\right)+l_{1}l_{3}m_{3}{\ddot{q}}_{3}\cos\left(q_{1}-q_{3}\right)\\ & -l_{1}l_{4}m_{5}{\ddot{q}}_{4}\cos\left(q_{1}-q_{4}\right)-l_{1}l_{c2}m_{2}{\ddot{q}}_{2}\cos\left(q_{1}-q_{2}\right)-l_{1}l_{c3}m_{3}{\ddot{q}}_{3}\cos\left(q_{1}-q_{3}\right)\\ & -l_{1}l_{c4}m_{4}{\ddot{q}}_{4}\cos\left(q_{1}-q_{4}\right)-l_{1}l_{c5}m_{5}{\ddot{q}}_{5}\cos\left(q_{1}-q_{5}\right) \end{array}$$

(A15)
$$\begin{array}{cc} \tau_{2}= & I_{2}{\ddot{q}}_{2}+{l_{2}}^{2}m_{2}{\ddot{q}}_{2}+{l_{2}}^{2}m_{3}{\ddot{q}}_{2}+{l_{2}}^{2}m_{4}{\ddot{q}}_{2}+{l_{2}}^{2}m_{5}{\ddot{q}}_{2}+{l_{c2}}^{2}m_{2}{\ddot{q}}_{2}\\ & -2l_{2}l_{c2}m_{2}{\ddot{q}}_{2}-gl_{2}m_{2}\sin\left(q_{2}\right)-gl_{2}m_{3}\sin\left(q_{2}\right)-gl_{2}m_{4}\sin\left(q_{2}\right)\\ & -gl_{2}m_{5}\sin\left(q_{2}\right)+gl_{c2}m_{2}\sin\left(q_{2}\right)+l_{1}l_{2}m_{2}{\ddot{q}}_{1}\cos\left(q_{1}-q_{2}\right)\\ & +l_{1}l_{2}m_{3}{\ddot{q}}_{1}\cos\left(q_{1}-q_{2}\right)+l_{1}l_{2}m_{4}{\ddot{q}}_{1}\cos\left(q_{1}-q_{2}\right)+l_{1}l_{2}m_{5}{\ddot{q}}_{1}\cos\left(q_{1}-q_{2}\right)\\ & +l_{2}l_{3}m_{3}{\ddot{q}}_{3}\cos\left(q_{2}-q_{3}\right)-l_{2}l_{4}m_{5}{\ddot{q}}_{4}\cos\left(q_{2}-q_{4}\right)-l_{1}l_{c2}m_{2}{\ddot{q}}_{1}\cos\left(q_{1}-q_{2}\right)\\ & -l_{2}l_{c3}m_{3}{\ddot{q}}_{3}\cos\left(q_{2}-q_{3}\right)-l_{2}l_{c4}m_{4}{\ddot{q}}_{4}\cos\left(q_{2}-q_{4}\right)-l_{2}l_{c5}m_{5}{\ddot{q}}_{5}\cos\left(q_{2}-q_{5}\right)\\ & -l_{1}l_{2}m_{2}{{\dot{q}}_{1}}^{2}\sin\left(q_{1}-q_{2}\right)-l_{1}l_{2}m_{3}{{\dot{q}}_{1}}^{2}\sin\left(q_{1}-q_{2}\right)-l_{1}l_{2}m_{4}{{\dot{q}}_{1}}^{2}\sin\left(q_{1}-q_{2}\right)\\ & -l_{1}l_{2}m_{5}{{\dot{q}}_{1}}^{2}\sin\left(q_{1}-q_{2}\right)+l_{2}l_{3}m_{3}{{\dot{q}}_{3}}^{2}\sin\left(q_{2}-q_{3}\right)-l_{2}l_{4}m_{5}{{\dot{q}}_{4}}^{2}\sin\left(q_{2}-q_{4}\right)\\ & +l_{1}l_{c2}m_{2}{{\dot{q}}_{1}}^{2}\sin\left(q_{1}-q_{2}\right)-l_{2}l_{c3}m_{3}{{\dot{q}}_{3}}^{2}\sin\left(q_{2}-q_{3}\right)-l_{2}l_{c4}m_{4}{{\dot{q}}_{4}}^{2}\sin\left(q_{2}-q_{4}\right)\\ & -l_{2}l_{c5}m_{5}{{\dot{q}}_{5}}^{2}\sin\left(q_{2}-q_{5}\right) \end{array}$$

(A16)
$$\begin{array}{cc} \tau_{3}= & I_{3}{\ddot{q}}_{3}+{l_{3}}^{2}m_{3}{\ddot{q}}_{3}+{l_{c3}}^{2}m_{3}{\ddot{q}}_{3}-2l_{3}l_{c3}m_{3}{\ddot{q}}_{3}-gl_{3}m_{3}\sin\left(q_{3}\right)\\ & +gl_{c3}m_{3}\sin\left(q_{3}\right)+l_{1}l_{3}m_{3}{\ddot{q}}_{1}\cos\left(q_{1}-q_{3}\right)+l_{2}l_{3}m_{3}{\ddot{q}}_{2}\cos\left(q_{2}-q_{3}\right)\\ & -l_{1}l_{c3}m_{3}{\ddot{q}}_{1}\cos\left(q_{1}-q_{3}\right)-l_{2}l_{c3}m_{3}{\ddot{q}}_{2}\cos\left(q_{2}-q_{3}\right)-l_{1}l_{3}m_{3}{{\dot{q}}_{1}}^{2}\sin\left(q_{1}-q_{3}\right)\\ & -l_{2}l_{3}m_{3}{{\dot{q}}_{2}}^{2}\sin\left(q_{2}-q_{3}\right)+l_{1}l_{c3}m_{3}{{\dot{q}}_{1}}^{2}\sin\left(q_{1}-q_{3}\right)+l_{2}l_{c3}m_{3}{{\dot{q}}_{2}}^{2}\sin\left(q_{2}-q_{3}\right) \end{array}$$

(A17)
$$\begin{array}{cc} \tau_{4}= & I_{4}{\ddot{q}}_{4}+{l_{4}}^{2}m_{5}{\ddot{q}}_{4}+{l_{c4}}^{2}m_{4}{\ddot{q}}_{4}+gl_{4}m_{5}\sin\left(q_{4}\right)+gl_{c4}m_{4}\sin\left(q_{4}\right)\\ & -l_{1}l_{4}m_{5}{\ddot{q}}_{1}\cos\left(q_{1}-q_{4}\right)-l_{2}l_{4}m_{5}{\ddot{q}}_{2}\cos\left(q_{2}-q_{4}\right)-l_{1}l_{c4}m_{4}{\ddot{q}}_{1}\cos\left(q_{1}-q_{4}\right)\\ & -l_{2}l_{c4}m_{4}{\ddot{q}}_{2}\cos\left(q_{2}-q_{4}\right)+l_{4}l_{c5}m_{5}{\ddot{q}}_{5}\cos\left(q_{4}-q_{5}\right)+l_{1}l_{4}m_{5}{{\dot{q}}_{1}}^{2}\sin\left(q_{1}-q_{4}\right)\\ & +l_{2}l_{4}m_{5}{{\dot{q}}_{2}}^{2}\sin\left(q_{2}-q_{4}\right)+l_{1}l_{c4}m_{4}{{\dot{q}}_{1}}^{2}\sin\left(q_{1}-q_{4}\right)+l_{2}l_{c4}m_{4}{{\dot{q}}_{2}}^{2}\sin\left(q_{2}-q_{4}\right)\\ & +l_{4}l_{c5}m_{5}{{\dot{q}}_{5}}^{2}\sin\left(q_{4}-q_{5}\right) \end{array}$$

(A18)
$$\begin{array}{cc} \tau_{5}= & I_{5}{\ddot{q}}_{5}+{l_{c5}}^{2}m_{5}{\ddot{q}}_{5}+gl_{c5}m_{5}\sin\left(q_{5}\right)-l_{1}l_{c5}m_{5}{\ddot{q}}_{1}\cos\left(q_{1}-q_{5}\right)\\ & -l_{2}l_{c5}m_{5}{\ddot{q}}_{2}\cos\left(q_{2}-q_{5}\right)+l_{4}l_{c5}m_{5}{\ddot{q}}_{4}\cos\left(q_{4}-q_{5}\right)\\ & +l_{1}l_{c5}m_{5}{{\dot{q}}_{1}}^{2}\sin\left(q_{1}-q_{5}\right)+l_{2}l_{c5}m_{5}{{\dot{q}}_{2}}^{2}\sin\left(q_{2}-q_{5}\right)-l_{4}l_{c5}m_{5}{{\dot{q}}_{4}}^{2}\sin\left(q_{4}-q_{5}\right) \end{array}$$
